# Efficacy of Lytic Phage Cocktails on *Staphylococcus aureus* and *Pseudomonas aeruginosa* in Mixed-Species Planktonic Cultures and Biofilms

**DOI:** 10.3390/v12050559

**Published:** 2020-05-18

**Authors:** Legesse Garedew Kifelew, Morgyn S. Warner, Sandra Morales, Nicky Thomas, David L. Gordon, James G. Mitchell, Peter G. Speck

**Affiliations:** 1 College of Science and Engineering, Flinders University, Bedford Park, SA 5042, Australia; jim.mitchell@flinders.edu.au (J.G.M.); peter.speck@flinders.edu.au (P.G.S.); 2 St Paul’s Hospital Millennium Medical College, Addis Ababa 1271, Ethiopia; 3 Infectious Diseases Unit, Queen Elizabeth Hospital, Woodville, SA 5011, Australia; morgyn.warner@sa.gov.au; 4 Discipline of Medicine, University of Adelaide, Adelaide, SA 5005, Australia; 5 AmpliPhi Australia Pty Ltd., Brookvale, NSW 2100, Australia; morales.sandra@gmail.com; 6 Basil Hetzel Institute for Translational Health Research, School of Pharmacy and Medical Sciences, University of South Australia, Adelaide, SA 5001, Australia; nicky.thomas@unisa.edu.au; 7 Department of Microbiology and Infectious Diseases, College of Medicine and Public Health, Flinders University, Bedford Park, SA 5042, Australia; d.gordon@flinders.edu.au

**Keywords:** phage cocktail therapy, biofilm, planktonic culture, fluorescence, efficacy, mixed-species culture, *Staphylococcus aureus*, *Pseudomonas aeruginosa*

## Abstract

The efficacy of phages in multispecies infections has been poorly examined. The *in vitro* lytic efficacies of phage cocktails AB-SA01, AB-PA01, which target *Staphylococcus aureus* and *Pseudomonas aeruginosa,* respectively, and their combination against their hosts were evaluated in *S. aureus* and *P. aeruginosa* mixed-species planktonic and biofilm cultures. Green fluorescent protein (GFP)-labelled *P. aeruginosa* PAO1 and mCherry-labelled *S. aureus* KUB7 laboratory strains and clinical isolates were used as target bacteria. During real-time monitoring using fluorescence spectrophotometry, the density of mCherry *S. aureus* KUB7 and GFP *P. aeruginosa* PAO1 significantly decreased when treated by their respective phage cocktail, a mixture of phage cocktails, and gentamicin. The decrease in bacterial density measured by relative fluorescence strongly associated with the decline in bacterial cell counts. This microplate-based mixed-species culture treatment monitoring through spectrophotometry combine reproducibility, rapidity, and ease of management. It is amenable to high-throughput screening for phage cocktail efficacy evaluation. Each phage cocktail, the combination of the two phage cocktails, and tetracycline produced significant biofilm biomass reduction in mixed-species biofilms. This study result shows that these phage cocktails lyse their hosts in the presence of non-susceptible bacteria. These data support the use of phage cocktails therapy in infections with multiple bacterial species.

## 1. Introduction

Wound infections, particularly chronic wounds such as occur in diabetic foot ulcers (DFUs), are often polymicrobial and frequently involve multidrug-resistant (MDR) bacterial pathogens [1,2]. A recent study found that 50% of the bacterial isolates recovered from DFU infections were MDR [3]. Polymicrobial wound infections have reportedly increased in the last decade and include MDR *S. aureus* and *P. aeruginosa,* which are frequently isolated and therapeutically challenging bacteria [4,5]. Both *S. aureus* and *P. aeruginosa* are associated with severe wound infections, including orthopedic infections [4,6], are often isolated together [5], and may occupy different parts of wounds [7]. Co-infections with *S. aureus* and *P. aeruginosa* usually result in worse patient outcomes than infections due to either pathogen alone [8,9]. In addition to their high level of antibiotic resistance, *S. aureus* and *P. aeruginosa* form *in vivo* biofilms that typically result in increased tolerance to antibiotics and contribute to bacterial virulence [8] and immune evasion [10].

Most chronic wound infections involve biofilms, which render antibiotic treatment less effective. Moreover, bacteria within biofilms exhibit altered metabolic properties as compared to planktonic bacteria, which reduces the efficacy of antibiotics in this setting [1,9,11,12]. Phages represent a potential alternative or adjunct therapy for infections with antibiotic-resistant bacteria [13]. However, the efficacy of phages in mixed-species bacterial infections has been incompletely examined, and the limited literature contains conflicting reports [14]. Lytic phages kill their bacterial host by lysis (bursting the infected bacterial cell to release progeny phages) [15]. The process of phage infection and subsequent self-replication in bacteria offers advantages over antibiotics: phages amplify themselves at the infection site provided there are susceptible bacterial hosts [16]. Phages are highly specific to the bacterial species they infect, an advantage over broadly active antimicrobials, as phages are not expected to disrupt a patients’ normal microflora. Phages are able to lyse biofilm forms of their host bacteria, such as those typically found in infected DFUs [16,17]. Some studies also suggest that both of these antimicrobial agents in combination are more effective in controlling pathogenic bacteria than either alone [18,19].

Difficulties in growing different bacterial species together *in vitro*, as in the case of *S. aureus* and *P. aeruginosa,* make the study of bacterial interactions and efficacy of antibiotic agents complicated [20,21]. *P. aeruginosa* mostly kills or outcompetes *S. aureus* in *in vitro* co-cultures [22,23]. Medium containing bovine serum albumin (BSA) is recommended to allow better growth of *S. aureus* in the presence of *P. aeruginosa* [24,25]. In this study, we used fluorescence spectrophotometry to evaluate the effectiveness of phage cocktails of *S. aureus* and *P. aeruginosa* under mixed-species planktonic cultures. The use of different fluorescent proteins-labelled bacterial species in mixed-species culture allows monitoring, in real-time, of phage treatment effects based on the detection of a decrease in fluorescence relative to the untreated controls. Fluorescence spectrophotometry is easy to handle, reproducible, and a rapid technique to evaluate treatment efficacy [26,27]. To confirm the spectrophotometry results, we carried out bacterial counts post-treatment, using selective agars. The efficacy of these phage cocktails was also examined in mixed-species biofilms using bacterial counts.

## 2. Materials and Methods

### 2.1. Bacterial Species

Clinical isolates of *S. aureus* (*n* = 4) and *P. aeruginosa* (*n* = 4) were randomly selected from isolates obtained from South Australia Pathology. These were among the isolates that showed strong susceptibility in spot test and 73–88% biofilm biomass reduction on single-species biofilm experiments because of the phage cocktail and its components treatment (data provided as Supplementary Table S1). Laboratory strains mCherry-labelled *S. aureus* KUB7 [28] and GFP-labelled *P. aeruginosa* PAO1 were generously donated by A/Prof. Heather Jordan of Mississippi State University, USA, and Dr. Nicky Thomas of University of South Australia, Australia, respectively. The fluorescent proteins in the laboratory strains are driven by constitutive promoters and do not need antibiotics to maintain fluorescence expression. Both strains were susceptible to all antibiotics they were exposed during the VITEK^®^ 2 test.

### 2.2. Phage Cocktails

The phage cocktails AB-SA01 and AB-PA01 were provided by AmpliPhi Biosciences Corporation (now Armata Pharmaceuticals, Inc.) (Los Angeles, CA, USA). AB-SA01 is a combination of three *Myoviridae* staphylococcal phages designated J-Sa-36, Sa-83, and Sa-87. The mean titer, presented in plaque-forming unit/millilitre—PFU/mL, was 9.3 log_10_ (PFU/mL) for J-Sa-36 and Sa-83, 9.0 log_10_ (PFU/mL) for Sa-87, and 9.1 log_10_ (PFU/mL) for the combined product AB-SA01 on *S. aureus* laboratory strains RN4220 and SA6538. AB-PA01 is a combination of Pa-193 and Pa-204 from *Myoviridae,* and Pa-222 and Pa-223 from *Podoviridae*. The titer was 10.5 log_10_ (PFU/mL) for Pa-193, Pa-204, and Pa-222, 9.5 log_10_ (PFU/mL) for Pa-223, and 10.3 log_10_ (PFU/mL) for AB-PA01 on *P. aeruginosa* laboratory strains PAO1 and PA10145. The phage titre was determined using plaque assay, as described [29,30]. None of these phage components encode any known bacterial virulence or antibiotic resistance genes, and all phages were considered to be strictly lytic [29,30]. The phages were produced following the current good manufacturing practice standard (cGMP) and approved by the US Food and Drug Administration as investigational new drugs [30,31].

### 2.3. Bacterial Identification

The isolates were identified using standard microbiology methods and confirmed by matrix-assisted laser desorption/ionization-time of flight mass spectrometry (MALDI-TOF MS) biotyping (BRUKER Pty. LTD., Melbourne, Victoria, Australia). Antibiotic susceptibility patterns were determined by VITEK^®^ 2 (bioMérieux Australia Pty Ltd., Sydney, New South wales, Australia).

### 2.4. Mixed-Species Planktonic Cultures and Phage Cocktail Treatment

Mixed-species planktonic cultures were performed following an established protocol [32] with few modifications indicated as follows. In the case of fluorescently-labelled laboratory strains, 2-3 colonies from 18 h selective agar culture plates were suspended in sterile PBS and adjusted to 1.0 McFarland equivalence turbidity standard, containing approximately 8.5 log_10_ (CFU/mL). Each bacterial species suspension was diluted at 1:100 *v/v* with nutrient broth (Sigma-Aldrich, Sydney, New South wales, Australia) supplemented with 5% BSA (Sigma-Aldrich, Sydney, New South wales, Australia) and incubated for 2 h at 37 °C. The two bacterial suspensions were then mixed at the ratio of 1:3 *v/v* of GFP *P. aeruginosa* PAO1 to mCherry *S. aureus* KUB7 in a sterile 10 mL test tube. Two hundred microlitre triplicates of each mixture were transferred to a clear 96-well flat-bottom Greiner CELLSTAR^®^ polystyrene tissue culture plate (Sigma-Aldrich, Sydney, New South wales, Australia), and respective treatments were applied.

Mixed-species planktonic cultures were treated with one of (i) *S. aureus* phage cocktail AB-SA01; (ii) *P. aeruginosa* phage cocktail AB-PA01; (iii) a mixture of the two phage cocktails, AB-SA01+AB-PA01; (iv) gentamicin (positive control); or (v) PBS (negative control). The effect of each phage cocktail and the combination of the two phage cocktails was compared to the gentamicin- and PBS-treated groups.

AB-SA01, AB-PA01, or AB-SA01+AB-PA01 phage cocktails were applied at a multiplicity of infection (MOI) of one to fluorescently labelled *S. aureus-P. aeruginosa* mixed-species culture. Gentamicin was used as a positive control at 16 μg/mL as the minimum inhibitory concentration (MIC) to these isolates was ≤ 8 μg/mL. An equal volume of PBS to phage solutions was applied to negative control groups. A plate cover was applied, and the plate was wrapped with aluminium foil from the top and sides. The plate was incubated in a CLARIOstar Omega plate reader (BMG LABTECH Pty. Ltd., Melbourne, Victoria, Australia) for 24 h at 37 °C with 100 rpm constant double orbital shaking between measurements as described [33] for fluorescent protein-labelled strains. Fluorescence of mCherry and GFP was measured (in relative fluorescence unit, RFU) every 30 min in each well. The excitation and emission wavelengths were set at 570-15 and 620-20 nm for mCherry, and 470-15 and 515-20 nm for GFP detection, respectively. Signals from triplicate wells were averaged and corrected for blank wells containing only nutrient broth. After 24 h incubation, bacterial colony counts were performed using serial dilution on selective agars for each species, vancomycin-supplemented MacConkey for *P. aeruginosa,* and mannitol salt agar (MSA) for *S. aureus*. The experiment was repeated three times with the same protocol on different days. A similar protocol was followed for clinical isolates, except that incubation was in a standard incubator.

### 2.5. In Vitro Mixed-Species Biofilm Development and Phage Cocktail Treatment

Mixed-species biofilm development and treatment was conducted using a previously described procedure [34,35] with few modifications. Briefly, 2-3 colonies of 18 h culture of each isolate were independently suspended in sterile PBS and adjusted to 1.0 McFarland turbidity standard. These suspensions were pooled at 1:3 *v/v* ratio of *P. aeruginosa* to *S. aureus*, and 100 µL of the mixed suspension was transferred to 10 mL 5% BSA-nutrient broth. The final suspension was supplemented with 1% sterile glucose to facilitate biofilm development. Two hundred microlitres of the suspension was transferred in triplicate into a tissue culture plate and incubated for 48 h at 37 °C with 70 rpm agitation.

The treatment categories of mixed-species biofilms were (i) *S. aureus* phage cocktail AB-SA01; (ii) *P. aeruginosa* phage cocktail AB-PA01; (iii) a mixture of the two phage cocktails, AB-SA01+AB-PA01; (iv) tetracycline (positive control); and (v) PBS (negative control). The effect of each phage cocktail and a combination of the two phage cocktails was compared to the tetracycline- and PBS-treated groups. Tetracycline (Sigma-Aldrich Corporation, Sydney, New South wales, Australia) was used as a positive control in mixed-species biofilm treatment as it was strongly effective (*p* < 0.001), compared with PBS treatment, in biofilm biomass reduction on single-species biofilm treatment of both *S. aureus* and *P. aeruginosa* isolates. However, gentamicin did not produce significant biofilm biomass reduction (*p* > 0.05), compared with PBS treatment (Unpublished data). Tetracycline was not used as a positive control in mixed-species planktonic culture treatment experiments to avoid exaggerated fluorescence detection because of its color and fluorescent nature [36].

Next, the liquid culture was removed, and plates were washed gently twice using sterile deionized water. Then, 225 μL of AB-SA01, AB-PA01, or AB-SA01+AB-PA01 in the nutrient broth was applied to the respective treatment group biofilms. An equivalent volume of tetracycline and PBS to phage solution in nutrient broth were also applied as controls. The concentration of tetracycline was 128 µg/mL because *P. aeruginosa* isolates are susceptible to a higher concentration of tetracycline [37,38]. The MIC of tetracycline for *S. aureus* was ≤ 8 µg/mL during VITEK^®^ 2 antimicrobial susceptibility test. Treated biofilms were incubated for 12 h at 37 °C with no agitation. The biofilm was washed twice using 250 μL sterile PBS through careful pipetting. The biofilm-associated cells attached to the well surface were collected with 225 μL nutrient broth by pipetting after scraping the surface with a loop as described earlier [39,40]. After homogenization with a vortex mixer, the cell suspension was serially diluted, 10^−1^–10^−8^, in filter-sterilized 10 mM ferrous ammonium sulphate (FAS) supplemented nutrient broth to inactivate free phage [41] and incubated at room temperature for 15 min.

### 2.6. Viable Bacterial Cell Count

One hundred microlitres of the bacterial suspension from each serial dilution was then mixed with 3 mL nutrient soft agar warmed at 42 °C and dispensed over 37 °C pre-warmed MSA and vancomycin-supplemented MacConkey agar in triplicate and incubated at 37 °C for 24 h. Plates with approximately 30–300 colonies were taken from one of the dilutions, and colony count was carried out as previously described [42]. The bacterial cell count was calculated using the formula B = N/d where B = number of bacteria; N = average number of colonies counted on three plates; d = dilution factor as described earlier [43]. The results are expressed as logarithm-transformed values (log (CFU/mL)).

### 2.7. Statistical Analysis

STATA version 16 software was used for statistical analysis. Data are reported in terms of the mean. A comparison of experimental groups was performed using a one-way analysis of variance (two-tailed) or paired ‘*t-test*’. A *p* < 0.05 value was considered statistically significant.

## 3. Results

### 3.1. Effect of Phage Cocktails on Fluorescently Labelled Mixed-Species Planktonic Cultures

**Figure 1:**
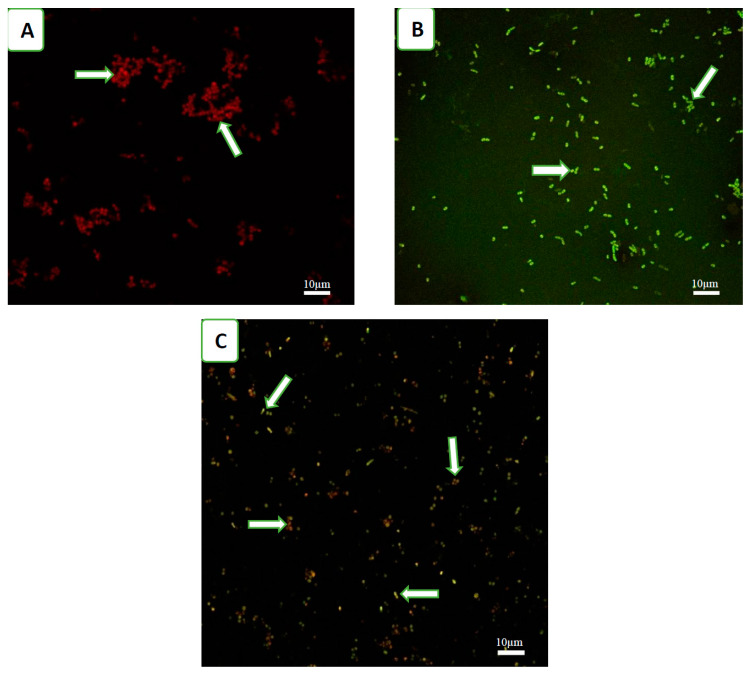
Fluorescence strain under confocal microscopy: (**A**) mCherry-labelled *S. aureus* KUB7, (**B**) GFP-labelled *P. aeruginosa* PAO1, and (**C**) mCherry-labelled *S. aureus* KUB7 mixed with GFP-labelled *P. aeruginosa* PAO1.

The fluorescence of each species alone or together was confirmed under a confocal microscope, as shown in Figure 1.

**Figure 2:**
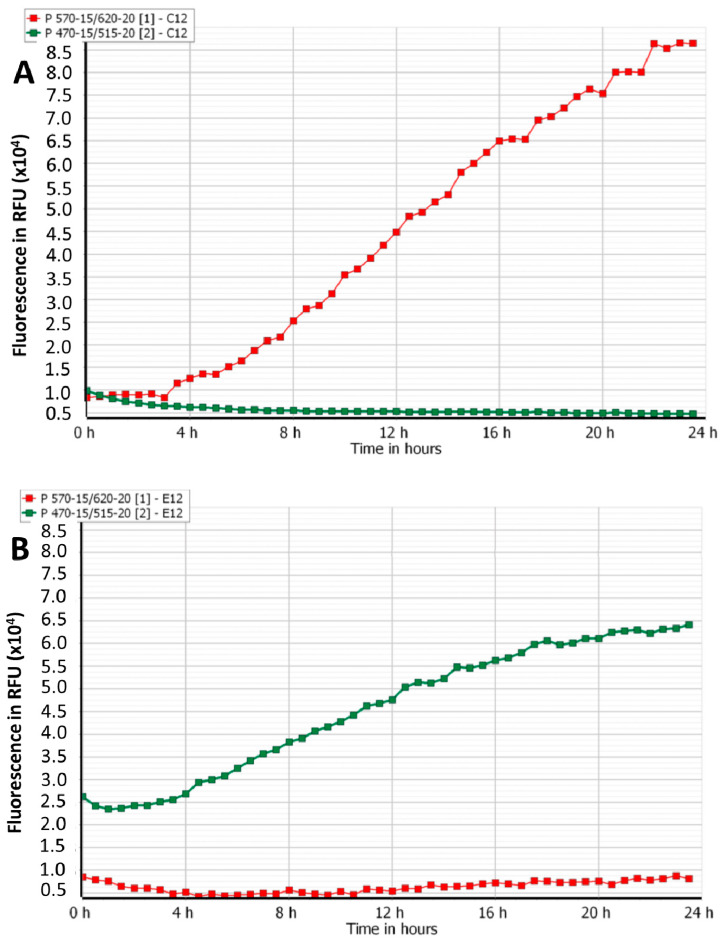

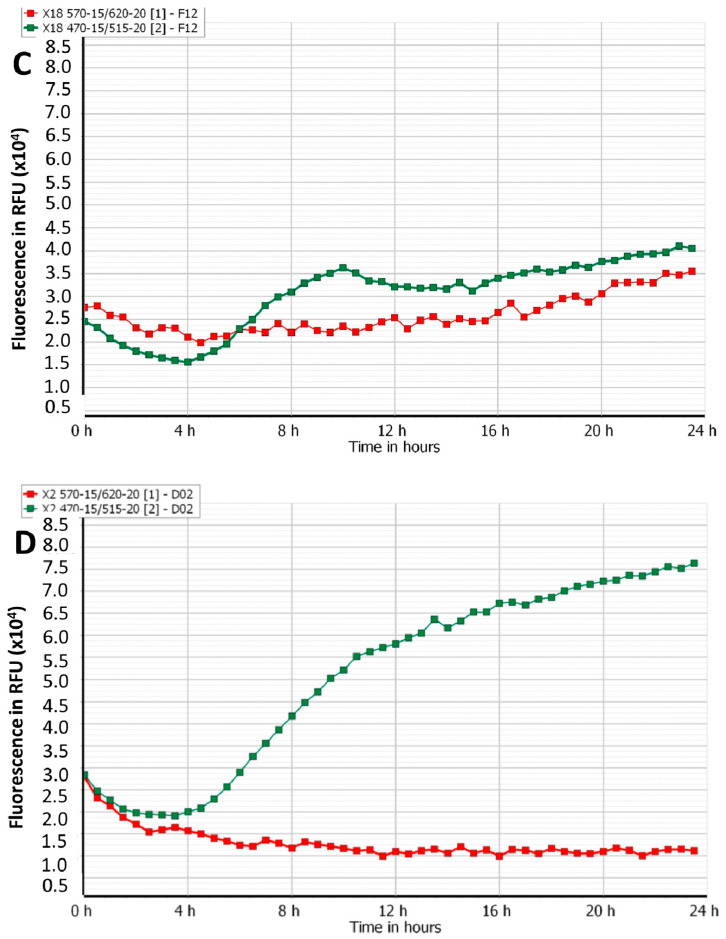

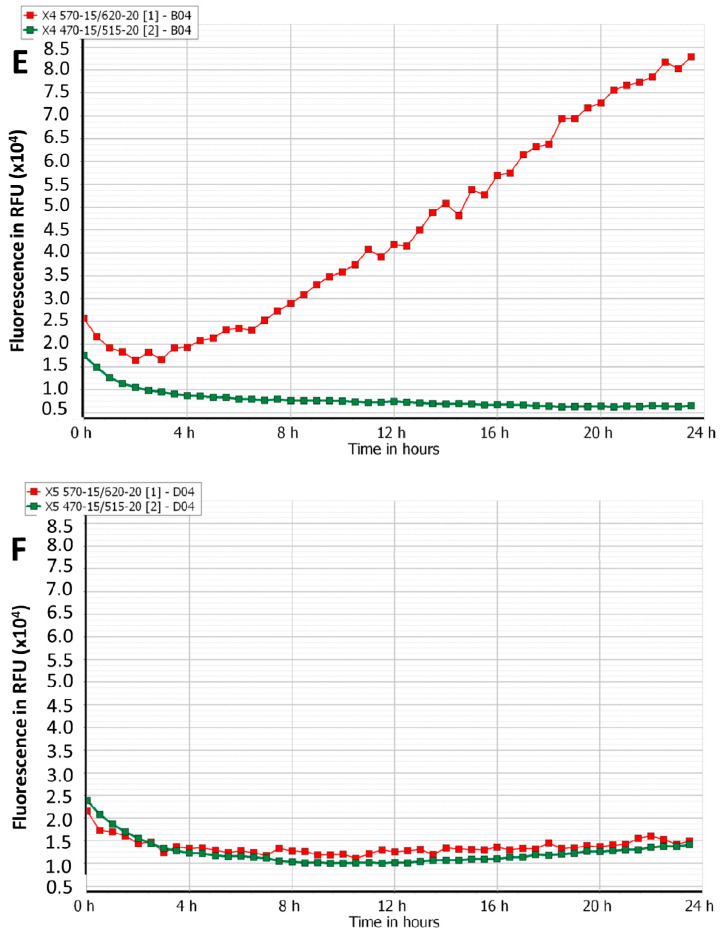

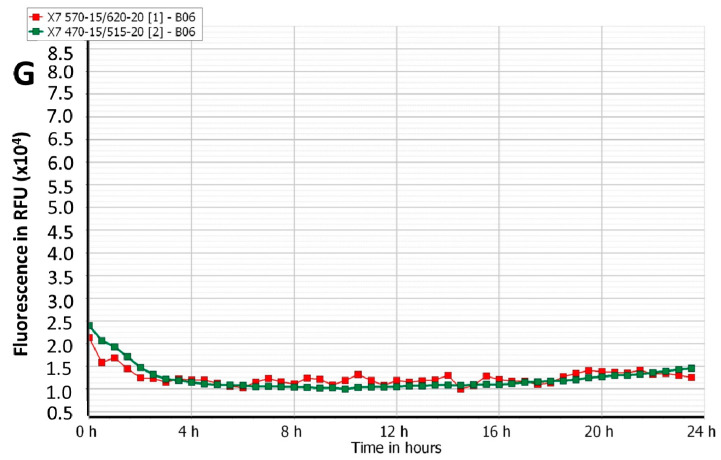
**A**–**G:** effect of phage cocktails on mCherry *S. aureus* KUB7 (red data points) and GFP *P. aeruginosa* PAO1 (green data points) single- and mixed-species planktonic cultures. RFU represents relative fluorescence unit. (**A**) PBS-treated mCherry *S. aureus* KUB7 (in red) single-species culture; the green graph is due to background detection since the machine was set for mCherry and GFP detection. (**B**) PBS-treated GFP *P. aeruginosa* PAO1 (in green) single-species culture; the red graph is due to background detection since the machine was set for mCherry and GFP detection. (**C**) PBS-treated mCherry *S. aureus* KUB7 and GFP *P. aeruginosa* PAO1 mixed-species culture. (**D**) AB-SA01-treated mCherry *S. aureus* KUB7 and GFP *P. aeruginosa* PAO1 mixed-species culture. (**E**) AB-PA01-treated mCherry *S. aureus* KUB7 and GFP *P. aeruginosa* PAO1 mixed-species culture. (**F**) AB-PA01+AB-SA01-treated mCherry *S. aureus* KUB7 and GFP *P. aeruginosa* PAO1 mixed-species culture. (**G**) Gentamicin-treated mCherry *S. aureus* KUB7 and GFP *P. aeruginosa* PAO1 mixed-species culture.

The growth of fluorescently-labelled *S. aureus* and *P. aeruginosa* in single- and mixed-species planktonic cultures in the presence and absence of phages is shown in Figure 2A–G. Compared to the phosphate-buffered saline (PBS) treatment, AB-SA01 and AB-PA01 treatments effectively halted the growth of their host throughout the 24 h follow-up period. In the single-species cultures treated with PBS, there was a marked increase in fluorescence of mCherry *S. aureus* KUB7 and GFP *P. aeruginosa* PAO1, as shown in Figure 2A,B, indicating bacterial growth. In the mixed-species cultures without phages, the magnitude of fluorescence slowly increased with time (Figure 2C). However, the maximum red and green fluorescence obtained was much lower than the fluorescence detected during single-species PBS-treated cultures (Figure 2A,B), suggesting that the mixed-species exhibited competition for nutrients or co-inhibitory effects. In mixed-species cultures treated with a single phage cocktail, the fluorescence of the target host was almost eliminated, while the non-target host was unaffected, as shown in Figure 2D,E, with fluorescence similar to PBS-treated single-species control.

When both phage cocktails were added to the mixed-species cultures, there was low fluorescence of both bacterial species, as shown in Figure 2F, similar to the inhibitory effect of gentamicin (Figure 2G), indicating that phage efficacy is not affected by the presence of non-host bacteria or other phages. The highest magnitude of red fluorescence in mCherry *S. aureus* KUB7 was detected from the untreated single-species culture (Figure 2A). In the case of GFP *P. aeruginosa* PAO1, the highest green fluorescence was detected from mixed-species culture treated with *S. aureus* phage cocktail, AB-SA01 (Figure 2D). The lowest magnitude of fluorescence from the target host in the mixed-species culture was observed when treated with each phage cocktail, as shown in Figure 2D,E. As expected, AB-SA01 and AB-PA01 exhibited no lytic effect on non-susceptible hosts (Figure 2D,E). While the fluorescence detected from a non-susceptible host showed an increase through time, the fluorescence obtained from the susceptible host remained low.

The decreases in fluorescence from each phage cocktail-, combinations of the two phage cocktails-, and gentamicin-treated groups were significantly lower compared to the PBS-treated group. The corresponding colony count results for each treatment group after 24 h are shown in Table 1 and confirm the results obtained with the fluorescence detection methodology.

### 3.2. Efficacy of Phage Cocktails on Laboratory and Clinical Isolates Mixed-Species Planktonic Cultures

The population of each bacterial species in mixed-species planktonic cultures at the end of 24 h of treatment was assessed. Compared to PBS-treated samples, AB-SA01- and AB-PA01-treated samples produced 3.3 log_10_ (CFU/mL) and 5.1 log_10_ (CFU/mL) reduction on their hosts, respectively, as shown in Table 1. These reductions in bacterial cell count were associated with the susceptibility of each bacterial isolates to the specific phage cocktail and its component phages during spot test (see complementary data, Table S1). When the same samples were treated with the combination of the two phage cocktails, AB-SA01+AB-PA01, the mean cell count of *S. aureus* and *P. aeruginosa* reduced by 4.7 log_10_ (CFU/mL) and 3.8 log_10_ (CFU/mL), respectively. The cell counts of one bacterial species showed an increase when the culture was treated with only a phage cocktail of the other species in the mixed-species culture. All planktonic cultures treated with gentamicin yielded no viable bacterial cells.

### 3.3. Effect of Phage Cocktails Treatment on Mixed-Species Biofilms

The findings of this study demonstrate that phage cocktails AB-SA01, AB-PA01, and a mixture of AB-SA01 and AB-PA01 successfully lysed their hosts in the presence of biofilms of non-susceptible species. These phage cocktails applied to *S. aureus* and *P. aeruginosa* mixed-species biofilms caused a statistically significant (*p* < 0.05; Table 2) reduction in the host cell population compared to the PBS-treated group, as shown in Table 2. However, the reduction of the cell population in *S. aureus* and *P. aeruginosa* was less than half of the decrease observed in planktonic culture treatment. Most of the tetracycline-treated cultures produced no or the lowest number of bacterial cells.

Compared to PBS treatment, the application of AB-SA01 or AB-PA01 alone did not produce a statistically significant effect on the cell count of the non-host bacterial species population (*p* > 0.05; 6.9 vs. 6.8 for AB-SA01 and 6.2 vs. 5.8 for AB-PA01, Table 2). The mean bacterial cell population of each species remained unaffected when treated with the other species’ phage cocktail alone. Treatment of mixed-species biofilms using the mixture of the two phage cocktails, AB-SA01+AB-PA01, produced similar cell reduction on both *S. aureus* and *P. aeruginosa* isolates as each phage cocktail treatment. The effect of tetracycline treatment caused a significant reduction in the population of both bacterial species.

## 4. Discussion

The rationale to examine the effect of phage treatment on mixed-species planktonic and biofilm cultures was that many wound infections are polymicrobial and contain bacteria in biofilm forms [44] and that there is a paucity of data on the action of phages under such circumstances. In polymicrobial infections, there exist interspecies interactions, ranging from antagonism to cooperation, that can significantly impact the pathogenicity of microbes and clinical outcomes of infections [45]. Examination of the bacterial population using fluorescence detection and CFU count results suggest that phage cocktail treatment is effective both in planktonic and biofilm states of the host bacteria under mixed-species cultures.

Multiple fluorescent proteins can be simultaneously applied to examine different microbial populations in real-time [46,47]. The use of mCherry in combination with GFP is suitable as the excitation and emission spectra of these proteins are well separated [46,48]. The advantages of using mCherry and GFP as markers include ease of detection, no exogenous substrate is required that may perturb biological samples, no need for cell processing for visualization, and they are suitable for real-time monitoring of cells in mixed cultures [49,50]. In this study, the high magnitude of fluorescence detected and the bacterial cell population obtained during single species culture without treatment show bacterial growth and fitness capability for the model while fluorescing [26,49,50,51]. Hence, we used mCherry *S. aureus* KUB7 and GFP *P. aeruginosa* PAO1 to distinguish them in mixed-species culture. In the current study, loss of mCherry or GFP fluorescence was considered an indication of bacterial death due to phage-induced lysis, which was supported by decreased or no CFU. Previous studies also showed that the decrease in fluorescence is due to cell death, suggesting it to be an early and sensitive marker of viability [26,52].

There was a strong association between the final bacterial density reading through fluorescence detection and bacterial cell population data as measured by colony count. For both mCherry *S. aureus* KUB7 and GFP *P. aeruginosa* PAO1, the fluorescence signal increased for PBS-treated or bacterial species unaffected by the treatment. The higher green fluorescence detected in AB-SA01-treated mixed-species culture than in GFP P. *aeruginosa* PAO1 single-species PBS-treated culture might be attributed to more pronounced *P. aeruginosa* population growth in the absence of competing organism or enhanced growth because of accessibility to more nutrients such as iron from the dead *S. aureus* cells [53]. The magnitude of fluorescence of mCherry or GFP obtained was minimal when the mixed-species bacterial cultures were treated with each phage cocktail alone. The fluorescence magnitude and bacterial cell population obtained during AB-SA01+AB-PA01 and gentamicin treatments were similar; the fluorescence records are low; the fluorescence graphs are overlapped and remained at their lower levels throughout the experiment period, and bacterial population recovered at the end of the experiment is minimal or none. This finding shows that treatment with each phage cocktails separately or in combination produced a similar effect to gentamicin treatment in planktonic mixed-species culture. Our experiments also demonstrated that bacterial density could be estimated, and the treatment effect evaluated in mixed-species cultures through fluorescence spectrophotometry using different fluorescent proteins-labelled bacterial species.

The magnitude of fluorescence detected in the untreated mixed-species culture was lower compared to untreated single-species cultures, which might be attributable to competition between the two bacterial species [21,22,23]. All these decreased or increased fluorescence detected were strongly associated with the decreased or increased colony count results, respectively. Our observations support previous reports that show the magnitude of fluorescence, absorbance, and colony count results are supplementary to one another in characterizing bacterial growth [54,55].

The magnitudes of fluorescence obtained from the three replica wells across the three independent experiments on different days with the same protocol were similar, demonstrating reliability of the method and reproducibility of results. The decrease in fluorescence of treated samples is consistent with efficient bacterial cell lysis by the host-specific phage cocktail as measured through colony count. This result agrees with a study that compared the fluorescence with a CFU count method in single-species culture [26]. In the mixed-species planktonic cultures of clinical isolates, treatment with each phage cocktail alone caused a significant (*p* < 0.001) decline of the target bacterial host population. Treatment with a combination of AB-SA01 and AB-PA01 also resulted in a significant (*p* < 0.05) decrease in cell density of both bacterial species. Our findings are similar to a study that showed planktonic *E. coli* grown in co-culture with *Salmonella* enterica did not survive attack from *E. coli* specific phages [56].

Mixed-species biofilms are complex communities that affect the physiological state of host bacterial cells and the availability of phage receptors, possibly due to competition with other bacterial species [14,39]. Our findings of mixed-species biofilm treatment show that the effect of both phage cocktails separately and in combination, AB-SA01+AB-PA01, significantly (*p* ≤ 0.05) reduced the target bacterial host population. The effect of these phage cocktails was lower in biofilm than in planktonic states of their hosts, which agrees with previous reports [35,39,57,58,59]. Possible explanations include: the complex extracellular biofilm matrix may reduce the efficacy of phages because of entrapment of phage particles [57], reduced multiplication of phages due to the large proportion of metabolically inactive host cells in biofilms [60], or shedding of phage receptors from the host bacteria in biofilms [61]. It might also be partly explained by the fact that dead cells resulting from phage attack might support surviving bacteria through serving as a nutrient reservoir and providing a shield from phage attack by phage binding to receptors on dead bacteria [62]. Tetracycline treatment showed superior bacterial population reduction, on both bacterial species, compared to the phage cocktail formulations used in this study. Our finding is consistent with a previous report that showed the application of phiIPLA-RODI, phiIPLA-C1C, and the combination of the two phages is more efficient in the planktonic phase than that in biofilms phase during *S. aureus* IPLA16 and *S. epidermidis* LO5081 mixed-species cultures [39].

In this study, we noted that phage cocktails applied to mixed-species biofilms could effectively reduce bacterial host populations. We did not observe the protection of susceptible bacterial hosts from phage attack in mixed-species biofilms by non-susceptible bacteria. This observation is consistent with some studies [34,40]. However, it is in contrast with a previous phage treatment study that concluded the structural heterogeneity of the biofilm from mixed-species produced pockets of unreachable susceptible bacteria [63]. Because we found significant bacterial host cell reduction during each phage cocktail treatment in mixed-species biofilm, protection from lysis for the target bacteria by the non-susceptible bacterial host cannot be assumed [40].

Our bacterial count data are in line with previous findings of biofilms infected with phages for 24 h and more extended periods [63,64]. Overall, our results confirm that lytic phages can be efficient in mixed-species planktonic and biofilm states. This study also demonstrates the feasibility of *in vitro* real-time monitoring of the efficacy of phage treatments using fluorescently labelled bacteria in mixed-species cultures through spectrophotometry, which is a simple, rapid, and reliable procedure.

## 5. Conclusions

Our findings suggest that the use of phage cocktails in mixed-species planktonic or biofilm state could provide practical alternatives to antibiotics in combating antibiotic-resistant infections. The association between the decrease or loss of fluorescence during real-time monitoring of the effect of phage cocktails with the decrease in numbers of bacterial cells as measured by colony counts, across the three replicas and three experiments in different days with the same protocol, shows the effectiveness of the phage cocktail treatments *in vitro* and repeatability of the results. The present study findings show that phages can reduce the host bacterial cell population significantly from planktonic and biofilm states. The lytic efficacy of AB-SA01, AB-PA01, and their combination on antibiotic-resistant bacterial hosts in mixed-species planktonic and biofilm phases is a clear indication of the potential of phages to mitigate antibiotic-resistant infections.

## Figures and Tables

**Table 1:** Bacteria cell count (log_10_ (CFU/mL)) of *S. aureus* and *P. aeruginosa* in mixed-species planktonic culture after 24 h phage cocktails, gentamicin, or PBS treatment.

Isolates Combination	Evaluated Isolate	Bacterial Cell Counts after Treatment				
		PBS	AB-SA01	AB-PA01	AB-SA01+ AB-PA01	Gentamicin
*S. aureus* KUB7 ^S^ and GFP PAO1 ^P^	*S. aureus* KUB7 ^S^	5.5	3.6	8.7	3.0	0
	GFP PAO1 ^P^	7.9	8.9	0	3.7	0
63-6538 ^S^ and 63-6598 ^P^	63-6538 ^S^	5.6	1.5	7.9	0	0
	63-6598 ^P^	5.3	6.0	0	0	0
63-2498 ^S^ and 63-5497 ^P^	63-2498 ^S^	6.2	0	6.6	0	0
	63-5497 ^P^	7.5	8.0	3.5	4.0	0
63-5656 ^S^ and 63-6036 ^P^	63-5656 ^S^	4.8	3.6	6.1	0	0
	63-6036 ^P^	6.6	8.1	3.3	4.2	0
Summarized treatment effect						
	*S. aureus* mean	5.5	2.2	7.3	0.8	0
	*S. aureus* reduction	---	3.3	+1.8	4.7	5.5
	*P. aeruginosa* mean	6.8	7.8	1.7	3.0	0
	*P. aeruginosa* reduction	---	+1.0	5.1	3.8	6.8

^S^*S. aureus* isolates, ^P^ *P. aeruginosa* isolates, + indicates an increase in bacterial count compared to PBS treatment.

**Table 2:** Bacteria count (log_10_ (CFU/mL)) of *S. aureus* and *P. aeruginosa* in mixed-species biofilms after 24 h phage cocktails, tetracycline, and PBS treatment.

Isolates Combination	Evaluated Isolate	Bacterial Cell Counts after Treatment				
		PBS	AB-SA01	AB-PA01	AB-SA01+ AB-PA01	Tetracycline
*S. aureus* KUB7 ^S^ and PAO1 GFP ^P^	*S. aureus* KUB7 ^S^	6.2	4.5	7.4	5.5	3.8
	PAO1 GFP ^P^	6.4	6.3	3.8	4.0	3.9
63-6538 ^S^ and 63-6598 ^P^	63-6538 ^S^	5.2	4.4	4.7	3.6	3.0
	63-6598 ^P^	5.5	5.6	3.6	3.7	0
63-2498 ^S^ and 63-5497 ^P^	63-2498 ^S^	6.3	4.4	5.2	5.5	0
	63-5497 ^P^	7.1	6.2	5.5	5.2	0
63-5656^S^ and 63-6036^P^	63-5656 ^S^	7	4.9	5.9	5.3	0
	63-6036 ^P^	8.5	8.9	4.7	6.3	0
Summarized treatment effect						
	*S. aureus* mean	6.2	4.6	5.8	5.0	1.7
	*S. aureus* reduction	----	1.6	0.4	1.2	4.5
	*P. aeruginosa* mean	6.9	6.8	4.4	4.8	1.0
	*P. aeruginosa* reduction	----	0.1	2.5	2.1	5.9

^S^*S. aureus* isolates, ^P^ *P. aeruginosa* isolates.

## Supplementary Materials

The following are available online at https://www.mdpi.com/1999-4915/12/5/559/s1, Table S1: Phage cocktail and component phages susceptibility and biofilm reduction (in percentage) after phage cocktail treatment of the selected bacterial isolates.
