# Supplementary material for: Efficacy of Lytic Phage Cocktails on Staphylococcus aureus and Pseudomonas aeruginosa in Mixed-Species Planktonic Cultures and Biofilms
Source: Viruses. 2020 May 18;12(5):559. doi: 10.3390/v12050559 (PMC7291191; doi:10.3390/v12050559)
Supplement: Supplementary file 1 [file viruses-12-00559-s001.pdf]

Table 3: Phage cocktail and component phages susceptibility and biofilm reduction (in percentage - %) after phage cocktail treatment of the selected bacterial isolates

No.	Bacterial isolate	Susceptibility to phages									% biofilm reduction	
		J-Sa-36	Sa-83	Sa-87	AB-SA01	Pa-93	Pa-204	Pa-222	Pa-223	AB-PA01	AB-SA01	AB-PA01
1.	*S. aureus* KUB7	S	S	S	S	n/a	n/a	n/a	n/a	n/a	72.84	n/a
2.	63-6538	S	S	S	S	n/a	n/a	n/a	n/a	n/a	87.66	n/a
3.	63-2498	S	S	S	S	n/a	n/a	n/a	n/a	n/a	80.00	n/a
4.	63-6565	S	S	S	S	n/a	n/a	n/a	n/a	n/a	75.68	n/a
5.	GFP PAO1	n/a	n/a	n/a	n/a	S	S	S	S	S	n/a	73.36
6.	63-6598	n/a	n/a	n/a	n/a	S	I	S	S	S	n/a	88.24
7.	63-5497	n/a	n/a	n/a	n/a	S	S	S	S	S	n/a	80.68
8.	63-6036	n/a	n/a	n/a	n/a	I	S	S	S	S	n/a	72.78

Key: S = Susceptible, I = Intermediate, n/a = Not applicable
